# Femoral neck locking plate versus multiple cannulated screws for femoral neck fractures in young adults: a randomized controlled trial

**DOI:** 10.1186/s12891-025-09019-7

**Published:** 2025-08-18

**Authors:** Yasser Ahmed Othman, Abdelrahman Hafez Khalefa, Islam Mohammed Ahmed, Khalaf Fathy Elsayed Ahmed

**Affiliations:** Orthopaedics and Traumatology Department, Sohag Faculty of Medicine, Sohag University, Sohag, Egypt

**Keywords:** Femoral neck fracture, Femoral neck locking plate, Multiple cannulated cancellous screws, Internal fixation, Functional recovery

## Abstract

**Background:**

Managing femoral neck fractures (FNFs) in young adults remains a significant clinical dilemma. No single internal fixation method has demonstrated clear superiority. The aim of this study was to compare the clinical and radiographic outcomes of FNFs in young adults treated with femoral neck locking plate (FNLP) or conventional partially-threaded 6.5 mm multiple cannulated cancellous screws (MCCS).

**Methods:**

A randomized controlled clinical trial (RCT) study was conducted on 74 patients to assess FNLP and MCCS in management of FNFs in young adults in Sohag university hospital between October 2022 and October 2024. The outcomes included Harris Hip Score (HHS), weight-bearing timelines, radiographic union times, and complication rates.

**Results:**

FNLP demonstrated superior functional outcomes with significantly higher HHS scores compared to MCCS. Patients treated with FNLP achieved earlier partial and full weight-bearing (*p* <.001) and faster radiographic union times (*p* =.012), indicating better biomechanical stability. MCCS had a significantly shorter operative time at (49.3 ± 3.5 min) compared to the FNLP group at (62.3 ± 9.9 min), (*p* =.042). Complication rates, including femoral neck shortening, avascular necrosis, and infection, were comparable between the two groups.

**Conclusion:**

FNLP is a more effective fixation method for young adults with FNFs, offering faster functional recovery and improved radiographic outcomes. MCCS demonstrated significant shorter operative time which is a potential advantage especially in resources-constrained settings. Complication rates were similar between FNLP and MCCS, making MCCS a viable option in selected cases based on fracture severity, surgical expertise, and resources availability.

**Level of evidence:**

Level II therapeutic: prospective randomized controlled clinical trial.

**Trial registration:**

The trial was retrospectively registered at 27 November, 2023 at www.clinicaltrials.gov (Trial Registration Number: NCT06162637).

**Supplementary Information:**

The online version contains supplementary material available at 10.1186/s12891-025-09019-7.

## Background

Femoral neck fractures (FNFs) are common injuries seen in the elderly patients due to trivial trauma and in young patients with high-energy trauma. Immediate diagnosis and management are required to prevent threatening joint complications [1–4].

Managing FNF in young adults remains a significant clinical dilemma [5–8]. The preservation of the native hip anatomy and biomechanics is essential for normal functions of hip in active young adults. Because of the vulnerable blood supply to the femoral head, there is a high risk of developing avascular necrosis (AVN) and non-union following these fractures [9–11].

Any sort of surgical fixation of FNFs should provide both preservation of the blood supply to the femoral head and enough mechanical stability until the fracture unites. Several methods for internal fixation of FNFs have been described including; multiple cannulated compression screws, locking plates of different types, dynamic hip screw (DHS) with an anti-rotational screw, and femoral neck system (FNS) [12–18]. No single internal fixation method has demonstrated clear superiority [19, 20].

The aim of this RCT study was to compare the clinical and radiographic outcomes of FNFs in young adults treated with femoral neck locking plate (FNLP) or conventional multiple cannulated cancellous screws (MCCS), aiming to determine the best method of fixation of this fracture.

We hypothesized that FNLP would show better functional and radiographic outcomes compared to MCCS in the treatment of FNFs in young adults.

## Methods

### Trial design

This parallel-group randomized controlled trial (RCT) was conducted at the orthopedics & trauma surgery department, Sohag university hospital (a level-one North African trauma center) between October 2022 and October 2024. Approval of the study from our institution’s Ethical Committee was obtained (Institutional review board (IRB) no: Soh-Med-23-11-02MS). It was retrospectively registered at 27 November, 2023 at ClinicalTrials.gov (NCT06162637). The study adhered to the CONSORT 2010 guidelines for reporting RCTs [21] (Supplementary material 1)

The study was carried out according to the declarations of Helsinki, and written informed consents were obtained from all patients.

### Sample size calculation

The required sample size was calculated based on our primary endpoint, the Harris Hip Score (HHS) at the final follow-up. Drawing on previous literature, we anticipated a clinically relevant difference of 6 points between the two groups, with a standard deviation of approximately 10. Using a two-sided significance level of 0.05 and aiming for 80% statistical power, the minimum sample size was determined to be 44 patients per group. We enrolled a total of 97 patients. Nonetheless, due to practical constraints related to recruitment and study duration, we were able to collect a total of 74 patients for final analysis, evenly distributed between the two treatment arms. While this slightly reduced the power of the study, we believe the sample remains sufficient to observe meaningful trends and draw clinically relevant conclusions.

### Participants (inclusion and exclusion criteria)

Participants included in the study were individuals aged 18–55 years, of any gender, who presented with unilateral traumatic femoral neck fractures and were independent walkers prior to the trauma without any aids. The injury had to have occurred within 7 days prior to treatment. Exclusion criteria were open fractures, pathological fractures, preexisting deformities or osteoarthritis, other pelvic or ipsilateral femoral injuries, renal impairment or ongoing high-dose steroid therapy, and neglected FNF. These criteria ensured the selection of a homogenous group suitable for assessing the specified interventions in the trial.

### Randomisation

#### Sequence generation

The random allocation sequence was generated by an independent statistician not involved in the clinical care or outcomes assessment of patients. A computer-generated random number table was used to assign the participants in a 1:1 ratio to either the FNLP group or the MCCS group.

#### Allocation concealment mechanism

Group assignments were kept in sequentially numbered, opaque, sealed envelopes prepared by the same independent statistician. This method ensured allocation concealment and prevented foreknowledge of group assignments.

#### Implementation

Participants were enrolled by a research coordinator who was not involved in the surgical procedures or outcome assessments. Upon enrollment and after confirming eligibility, the coordinator opened the next sequential envelope to determine the assigned intervention. Surgeons were informed of the allocation only after envelope opening to maintain allocation concealment up to the point of intervention.

#### Blinding

The study was open-labeled as the intervention techniques were visibly different, making it impractical to blind the surgeons or participants to the treatment type. Thus, no blinding was used during the intervention phase, which is typical for surgical trials where the type of surgery cannot be concealed.

The absence of complete blinding could have influenced several key outcomes. For example, the Harris Hip Score (HHS) includes subjective components such as pain and function, which may have been affected by patient’s expectations or the treating team’s knowledge of the intervention. Similarly, the decisions regarding the timing of weight-bearing progression could have been subconsciously influenced by the surgeon’s confidence in one fixation method over the other.

To reduce such bias in future studies, it would be advisable to implement assessor blinding where feasible. For instance, postoperative HHS and Visual Analogue Scale (VAS) evaluations could be conducted by independent outcome assessors unaware of the surgical techniques used. While complete blinding is not always achievable in surgical trials, incorporating blinded outcome assessment would strengthen the validity of the findings and mitigate observer and performance biases.

### Interventions

#### Preoperative assessment

All patients scheduled for surgery underwent a thorough pre-operative evaluation, which included collecting demographic data, medical history, and conducting a comprehensive clinical assessment with neurological and vascular evaluations. Routine laboratory tests such as CBC, liver and renal function tests were performed alongside essential radiological investigations, including X-rays in multiple views and pelvic CT scan. This evaluation aimed to ensure patients were adequately prepared for the upcoming surgical procedures. Fractures were classified according to Garden and Pauwels’ classifications [22, 23].

#### Surgical intervention

Operations were performed under spinal anesthesia with patients in a supine position on a fracture trauma table. All operations were carried out under the C-arm fluoroscopy. Prophylactic intravenous antibiotics were given to all patients within 30 min prior to surgery.

#### Fractures reduction

Fractures were reduced with gentle longitudinal traction and internal rotation, checked with image intensifiers in multiple views. Correction of rotational deformity and proper cortical alignment were achieved with internal rotation and adduction of the affected lower limb. Proper alignment and reduction were confirmed by ensuring the fracture was in anatomical or slight valgus alignment, with no extension or flexion on the lateral view. When necessary, an open approach to the femoral neck was employed to achieve reduction.

(A) Technique of femoral neck locking plate (FNLP).
  The FNLP procedure involved using a lateral approach.
  Once alignment was verified, a 5–8 cm lateral longitudinal incision near the greater trochanter allowed for the placement of the femoral neck locking plate. Soft tissue was carefully dissected to expose the femoral lateral cortex, where a K-wire might be used for maintaining fracture alignment.
  The plate was positioned centrally on the lateral femur cortex using an alignment jig. Starting with the apex of the triangle inferior screw hole; a 3.0 guide wire was inserted through the guiding sleeve along with the inferior third of the femoral neck, parallel to its axis, and ending in the subchondral zone. The central position of the guiding wire was checked on the lateral view. Another 2 proximal guide wires were inserted through guiding sleeves through the proximal 2 holes. Their position and length were checked using an image intensifier. Each locking 7.3 mm cannulated screw was inserted in turn and locked into the side plate. The plate was further fixed distally by one 4.5 mm distal screw into the femoral shaft. (Fig. 1)
  **Fig. 1:**
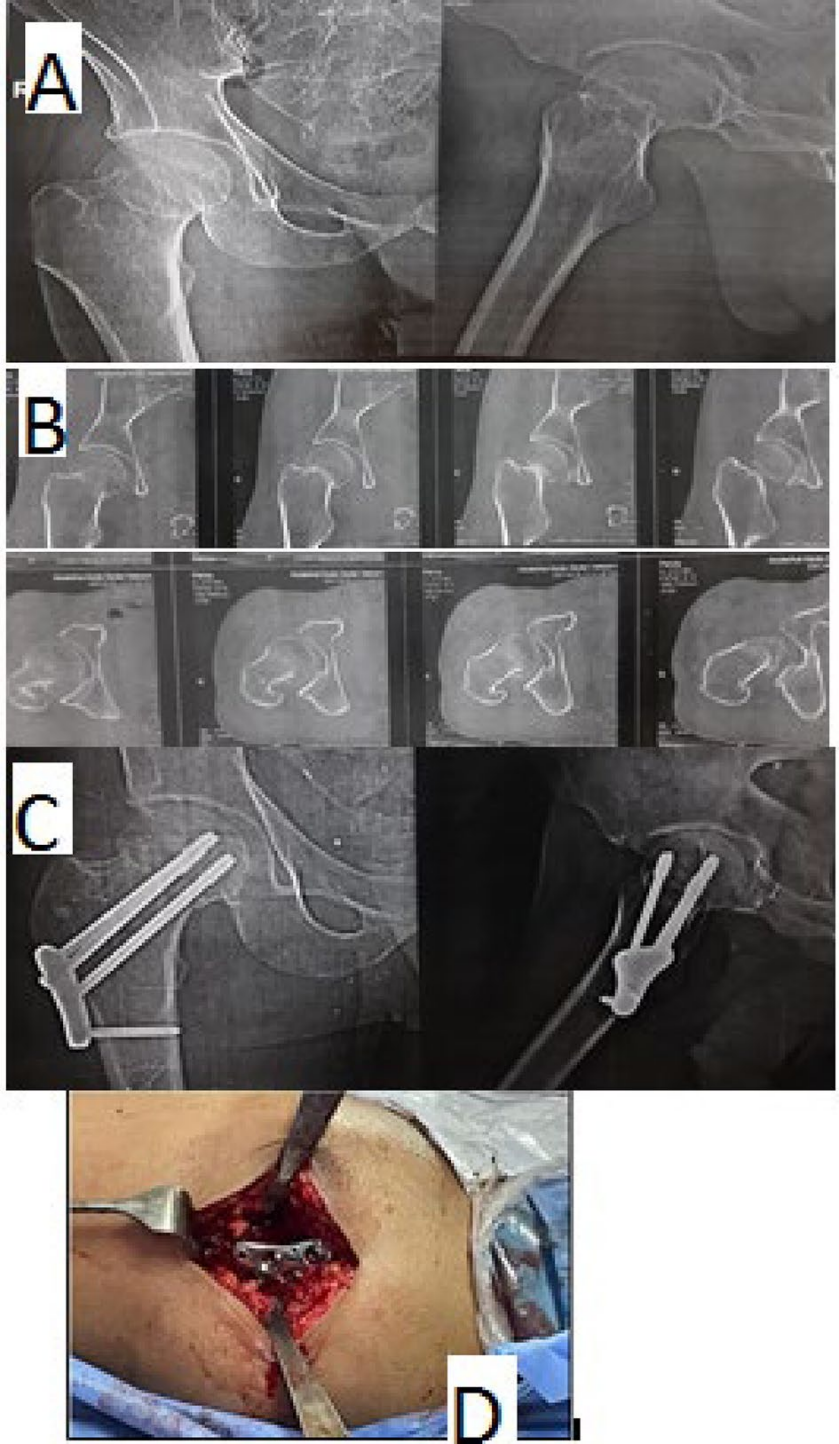
A 52 years old male patient with femoral neck fracture was resulting from road traffic accident and treated by FNLP: **A** Pre-operative plain x-rays showing subcapital femoral neck fracture of Garden type III and Pauwels type II. **B** CT scan. **C** immediate post-operative radiographs showing satisfactory reduction and fixation. **D** intra-operative clinical photo showing the surgical approach
(B) Technique of multiple cannulated cancellous screws (MCCS):
  The initial inferocentral guide wire was positioned just above the lesser trochanter level, visible in both views. A parallel guide was then used to accurately place the posterosuperior and anterosuperior guide wires. Screw length was determined by measuring the guide wire and subtracting 5 mm. Predrilling of the holes for cannulated screws by appropriate cannulated drill pit. Three 6.5 mm partially threaded cancellous cannulated screws were utilized. Washers were also used as space allowed. (Fig. 2)
  **Fig. 2:**
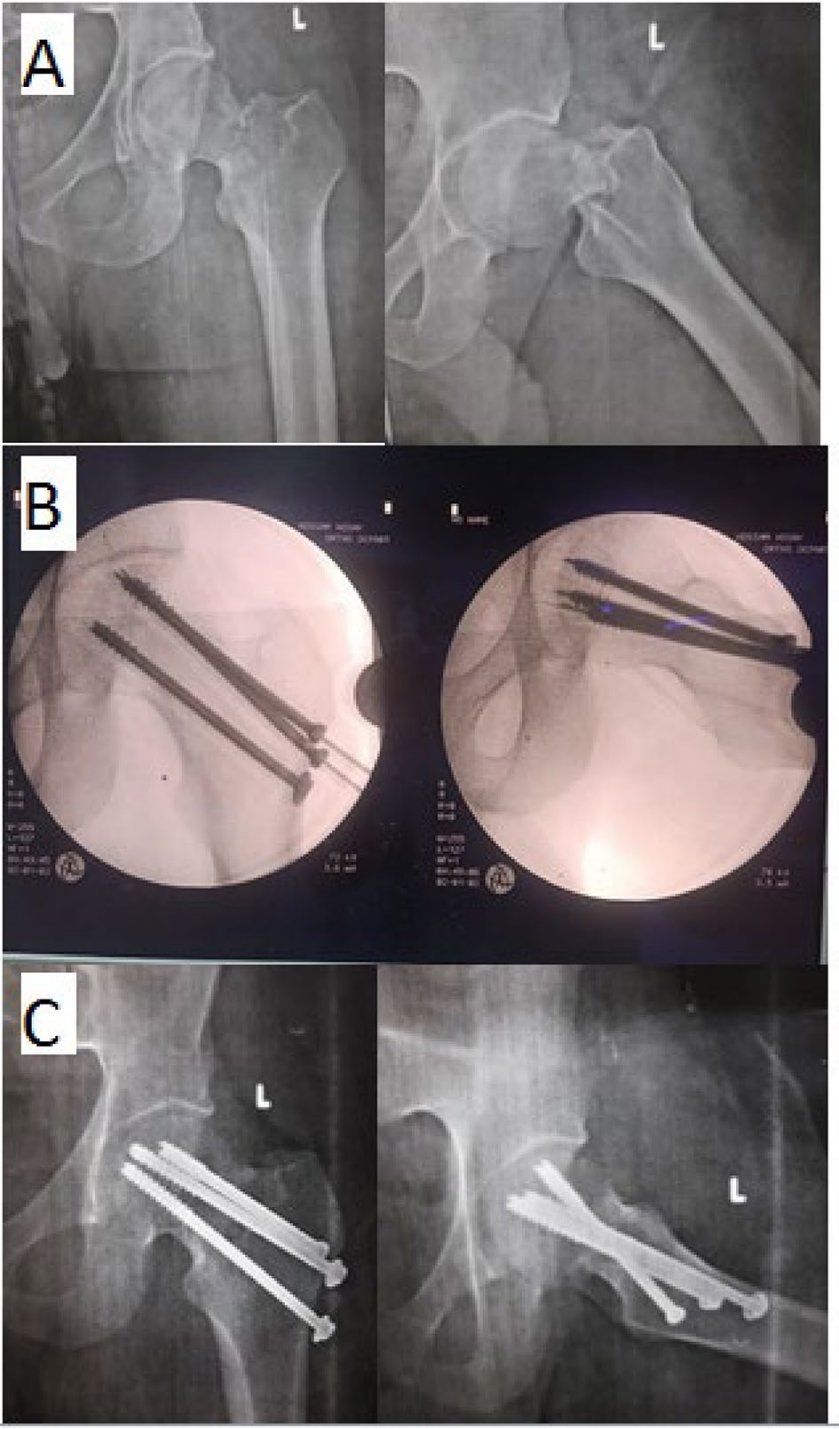
Thirty eight years old male patient with femoral neck fracture was resulting from falling from height and treated by MCCS. **A** Pre-operative plain x-rays showing transcervical femoral neck fracture of Garden type IV and Pauwels type III. **B** Intra-operative fluoroscopy showing confirmation of reduction and adequate fixation. **C** Immediate post-operative radiographs showing satisfactory reduction and fixation

#### Post-operative care

Postoperative management included immediate post-operative X-rays, IV antibiotics for three days, analgesics, and anticoagulants. Discharged within 1–2 days, patients were advised to use crutches without bearing weight for 6–12 weeks based on radiographic signs of fracture union. After 2 weeks stitches were removed.

#### Outcomes assessment

**Fig. 3:**
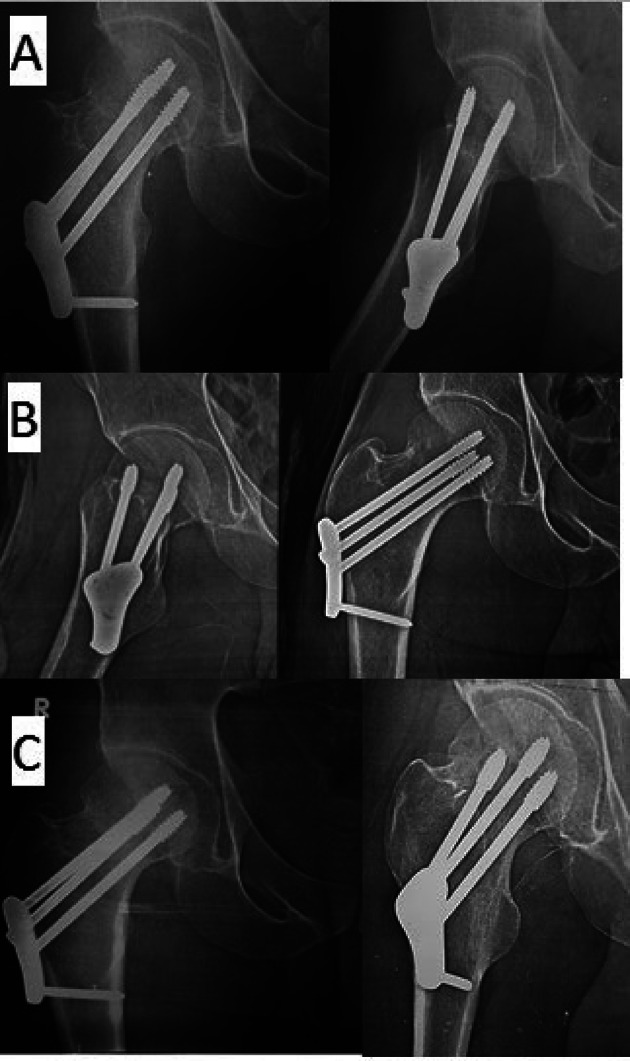
Follow up radiographs of the patient treated by FNLP. **A** X-rays at 2nd month post-operatively showing partial fracture healing. **B** Complete fracture healing and stable internal fixation at the 4th month postoperatively. **C** X-rays at 12th month follow up

**Fig. 4:**
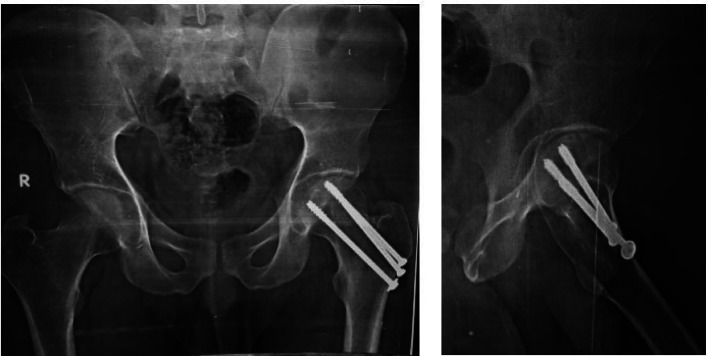
Follow up x-rays at 12th months of the patient treated by MCCS

Follow-up assessments at Sohag university hospitals occurred at 2, 6, 8, and 12 weeks, at monthly interval till healing, then at 12 months. Evaluations covered clinical aspects, such as weight-bearing time, pain (assessed using an 11-point VAS [24], range of motion, surgical site infections, and gait, alongside radiographic evaluations (Figs. 3 and 4). Radiographic follow-ups checked alignment, healing, and complications like avascular necrosis (AVN) and nonunion. The Harris Hip Score (HHS) was used for functional assessments at the final follow-up, categorizing outcomes from poor to excellent based on pain, function, deformity, and mobility [25].

### Statistical methods

Statistical analysis was done by SPSS v29 (IBM Inc., Chicago, IL, USA). Shapiro-Wilks’s test and histograms were used to evaluate the normality of the distribution of data. Quantitative parametric variables were presented as mean and standard deviation (SD) and compared between the two groups utilizing an unpaired Student’s t-test. Quantitative non-parametric data were presented as median and interquartile range (IQR) and were analyzed by Mann Whitney-test. Qualitative variables were presented as frequencies and percentages and were analyzed utilizing the Chi-square test or Fisher’s exact test when appropriate. A two-tailed P-value <.05 was considered statistically significant.

## Results

**Fig. 5:**
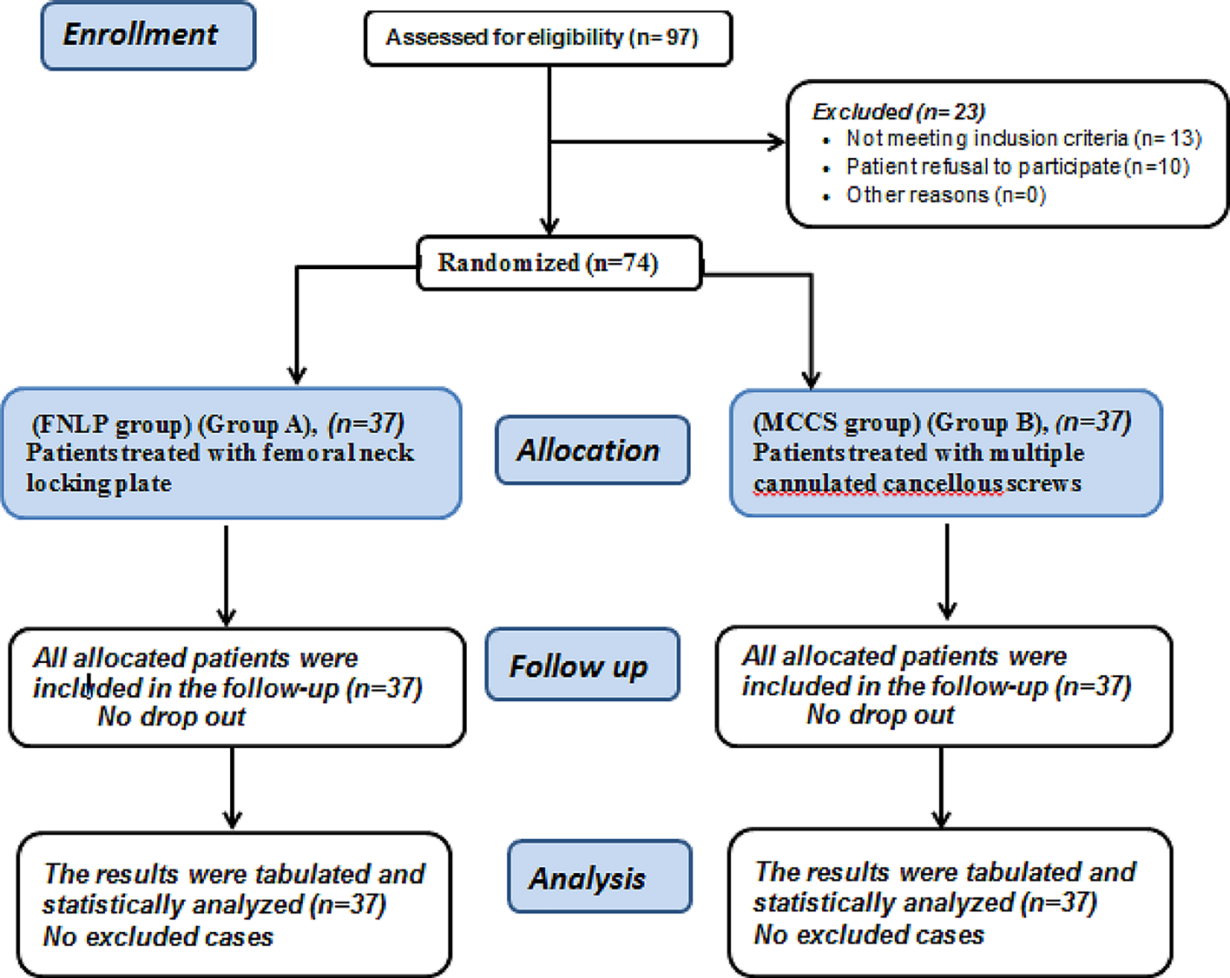
CONSORT flowchart of the enrolled patients

In this study, 97 patients were assessed for eligibility, 13 patients did not meet the criteria and 10 patients refused to participate in the study. The remaining patients were randomly allocated into two equal groups (37 patients in each); Group A: Patients underwent FNLP in treatment of FNFs, and Group B: Patients underwent MCCS in treatment of FNFs. All allocated patients were followed-up and analyzed statistically. (Fig. 5)

The mean operative time was significantly shorter in the MCCS group (49.3 ± 3.5 min) compared to the FNLP group (62.33 ± 9.9 min), (*p* =.042). No significant differences were found between the two groups as regard age, sex, side affected, mechanism of injury, Garden and Pauwels classifications of fractures, time lapsed between trauma and surgery, hospital stay, or follow up duration (Table 1).

**Table 1:** Baseline demographic and clinical characteristics of the two studied groups

Characteristics	FNLP group (Group A)	MCCS group (Group B)	*P* value
Total patients	37	37	
*Age at time of surgery (years)*			
Mean ± SD (range)	37.93 ± 10.82 (20–54)	40.07 ± 10.46 (25–55)	.587 (NS)
*Sex*			
Males, no (%)	28 (75.7%)	27 (73%)	.255 (NS)
Females, no (%)	9 (24.3%)	10 (27%)	
*Side affected*			
Right	15 (40.5%)	20 (54%)	.712 (NS)
Left	22 (59.5%)	17 (46%)	
*Mechanism of injury*			
Sports injury, no (%)	9 (24.3%)	7 (19%)	.820 (NS)
Road traffic accident, no (%)	20 (54%)	20 (54%)	
Falls, no (%)	8 (21.6%)	10 (27%)	
*Garden classification*			
II	10 (27%)	8 (21.6%)	.918 (NS)
III	12 (32.4%)	18 (48.6%)	
IV	15 (40.6%)	11 (29.8%)	
*Pauwels classification*			
I	6 (16.2%)	5 (13.5%)	.714 (NS)
II	12 (32.4%)	14 (37.8%)	
III	19 (51.4%)	18 (48.7%)	
*Time lapsed from trauma to surgery (days)*			
Mean ± SD (range)	1.23 ± 0.5 (1–3)	1.53 ± 0.6 (1–3)	.141 (NS)
*Operative time (minutes)*			
Mean ± SD (range)	62.33 ± 9.9 (45–75)	49.33 ± 3.52 (40–55)	.042 (S)
*Hospital stays (days)*			
Mean ± SD (range)	1.67 ± 0.5 (1–2)	1.61 ± 0.4 (1–2)	.132 (NS)
*Follow up period (months)*			
Mean ± SD (Range)	12.56 ± 2.49 (12–24)	12.44 ± 3.55 (12–24)	.449 (NS)

A. Functional outcomes
  At one year follow up; the Harris Hip Score (HHS) in the FNLP group A exhibited significantly better functional outcomes with a median score of 92 points compared to 86 points in the MCCS group (*p* =.008). Additionally, hip flexion was better in the FNLP group, averaging 115.23° versus 109.73° in the MCCS group, which was statistically significant (*p* =.003).
  There was no significant difference between two groups regarding VAS score at preoperative, one month postoperative and final follow up.
  The times to initiate partial and full weight-bearing were also significantly shorter in the FNLP group A, starting partial weight bearing at 6.07 weeks compared to 7 weeks in the MCCS group B, and achieving full weight bearing at 16.02 weeks versus 22.23 weeks, respectively (both *p* <.001) (Table 2).
  **Table 2:** Functional and radiographic outcomes of the two studied groups

Outcomes	FNLP group (Group A)		MCCS group (Group A)		*p* vales
	value	95% CI	value	95% CI	
Harris hip score at final follow up (points)					
Median (IQR)	92 (89–96)	**–**	86(80.5–92)	**–**	.008 (S)
Excellent, no (%)	19 (51.4%)	**–**	0 (0%)	**–**	.004 (S)
Good, no (%)	18 (48.6%)	**–**	26 (70.3%)	**–**	
Fair, no (%)	0 (0%)	**–**	11 (29.7%)	**–**	
Poor, no (%)	0 (0%)	**–**	0 (0%)	**–**	
*Range of motion of the hip, mean ± SD (range), (°)*					
Flexion	115.23 ± 3.47 (110–120)	(114-116.3)	109.73 ± 5.57 (101–120)	(107.9-111.5)	.003 (S)
Extension	12.33 ± 3.22 (10–15)	(11.3–12.4)	11.47 ± 3.31 (10–15)	(10.4–12.5)	.129
Abduction	35.73 ± 3.06 (30–40)	(34.7–36.7)	34.67 ± 3.89 (30–40)	(33.4–37.2)	.410
Rotation	36.33 ± 2.64 (32–40)	(35.5–37.8)	35.13 ± 3.25 (30–40)	(34.1–36.2)	.276
*VAS score for pain (points), mean ± SD (range)*					
Pre-op	5 ± 0.9 (5–8)	(4.7–5.3)	5 ± 0.5 (7–8)	(4.8–5.1)	.87 (NS)
1 month Post-op	2 ± 1.3 (2–4)	(1.6–2.4)	2 ± 0.9 (2–5)	(1.7–2.3)	.998 (NS)
Final follow up	1 ± 0.8 (0–2)	(0.7–1.25)	1 ± 0.9 (1–3)	(0.71–1.29)	.775 (NS)
*Time of partial weight-bearing (weeks), mean ± SD (range)*					
	6.07 ± 0.8 (6–8)	(5.81–6.32)	7 ± 0.9 (6–12)	(6.71–7.29)	<.001 (S)
*Time of full weight-bearing (weeks), mean ± SD (range)*					
	16.02 ± 1.66 (16–22)	(15.5–16.5)	22.23 ± 1.2 (22–30)	(21.8–22.6)	<.001 (S)
*Time of radiographic solid union (weeks), mean ± SD (range)*					
	15.2 ± 3.1 (12–20)	(14.2–16.2)	20.6 ± 2.64 (20–28)	(19.7-21.45)	.012 (S)

*CI *confidence interval
B. Radiographic outcomes
  The FNLP group showed a faster radiographic union, with an average time of 15.2 weeks compared to 20.6 weeks for the MCCS group (*p* =.012) (Table 2).
C. Complications
  Complications were insignificantly different between both groups (Table 3).
  **Table 3:** Complications of the two studied groups

Outcomes	FNLP group (Group A)	MCCS group (Group B)	*p* value
Femoral neck shortening	1 (2.7%)	3 (8.1%)	.753
Avascular necrosis	2 (5.4%)	1 (2.7%)	>.999
Non-union	1 (2.7%)	3 (8.1%)	.814
Peri-implant fracture	1 (2.7%)	1 (2.7%)	>.999
Infection	0 (0%)	1 (2.7%)	>.999
D. Factors affecting the outcomes
  Factors associated with excellent outcomes in FNLP group were younger age and less severity of fracture type according to Garden classification, (*p* =.048 &.026 respectively). Factors associated with good outcomes in MCCS group were male sex and less severity of fracture type according to Garden classification, (*p* =.046 &.049 respectively) (Table 4).
  **Table 4:** Factors affecting functional outcomes according to Harris hip score in the two studied groups

		FNLP group (Group A)			MCCS group (Group B)		*p* value
		Harris hip score		*p* value	Harris hip score		
		Excellent (*n* = 19)	Good (*n* = 18)		Good (*n* = 26)	Fair (*n* = 11)	
Age (years)^a^		32.14 ± 8.78	43 ± 10.25	.048^c^	39.92 ± 11.6	40.67 ± 5.13	.916^c^
Sex^b^	Male	13 (68.4%)	15 (83.3%)	.998^d^	19 (73%)	8 (72.7%)	.046^d^
	Female	6 (31.6%)	3 (16.7%)		7 (27%)	3 (27.3%)	
Side of injury^b^	Right	7 (36.84%)	8 (44.44%)	.314^d^	15 (57.7%)	5 (45.5%)	.876^d^
	Left	12 (63.16%)	10 (55.56%)		11 (42.3%)	6 (54.5%)	
Operative time (min)^a^		59.29 ± 9.76	62.25 ± 10.61	.718^c^	46.25 ± 3.11	48.67 ± 5.77	.862^c^
Time lapsed from trauma to surgery (days)^a^		0.9 ± 0.6	1.2 ± 0.4	.106^c^	1 ± 0.6	0.86 ± 0.37	.267^c^
Garden classification^b^	Type II	10 (52.6%)	0 (0%)	.026^d^	8 (30.8%)	0 (0%)	.049^d^
	Type III	6 (31.6%)	6 (33.33%)		12 (46.2%)	6 (54.5%)	
	Type IV	3 (15.8%)	12 (66.67%)		6 (23%)	5 (45.5%)	
Pauwels classification^b^	I	4 (21%)	2 (11.1%)	.977^d^	5 (19.3%)	0 (0%)	.697^d^
	II	8 (42%)	4 (22.2%)		6 (23%)	8 (72.7%)	
	III	7 (37%)	12 (66.7%)		15 (57.7%)	3 (27.3%)	
Mechanism of injury^b^	Sports injury	7 (36.8%)	2 (11.1%)	.389^d^	5 (19.2%)	2 (18.2%)	.251^d^
	Road traffic accidents	6 (31.6%)	14 (77.8%)		18 (69.3%)	2 (18.2%)	
	Falls	6 (31.6%)	2 (11.1%)		3 (11.5%)	7 (63.6%)	
Time of radiographic solid union (weeks)		14.86 ± 3.02	15.5 ± 3.34	.704^c^	18.3 ± 3.44	19.7 ± 2.9	.914^c^
Time of full weight bearing (weeks)		16.29 ± 1.8	18.38 ± 1.51	.305^c^	21.26 ± 2.2	24.7 ± 3.2	.624^c^

^a^Data are presented as mean ± standard deviation

^b^Data are presented as No (%)

^c^One-way ANOVA

^d^Chi-square test

## Discussion

This RCT study assessed the effectiveness of two fixation techniques, FNLP and MCCS, in treatment of FNFs in young adults. The major findings of this study at final follow up were the clinical and radiographic superiority of FNLP over MCCS in the treatment of FNFs in young adults with comparable complications rates between both techniques.

There was no statistically significant difference in the baseline demographic and clinical characteristics between both groups except for significantly shorter operative time in the MCCS group (49.3 ± 3.5 min) compared to the FNLP group (62.33 ± 9.9 min), (*p* =.042). These findings were supported by different studies which compared femoral neck locking plates to cannulated compression screws for treatment of FNFs in young adults as those of Wang et al. [14], Shu et al. [13], and Warschawski et al. [26]. Significant shorter operative time in MCCS group in the current study has advantages of reduced anesthesia exposure and resources utilization, especially in resources-constrained settings.

Regarding functional outcomes of this study; FNLP showed functional superiority to MCCS, with a significantly higher median HHS score (*p* =.008), supported by categorical distribution analysis (*p* =.004), suggesting better biomechanical stability. However, VAS scores for pain didn’t significantly differ between both groups either pre-operatively, one month post-operatively, or at final follow up (*p =*.871,.998 &.775 respectively). Wang et al. [14] conducted RCT to compare proximal femoral cannulated screw locking plate (CSLP) to multiple cancellous screws (MCS) in the treatment of displaced intra-capsular hip fractures (ICHFs) in 68 young adult patients. After a mean follow up period of 21.7 months for CSLP group, and 24.8 months for MCS group, there were no statistically significant differences in the HHS at one year after surgery between the two groups of patients (*p* =.092). They concluded that the use of CSLP in treatment of displaced ICHFs can reduce the rates of postoperative nonunion and overall complications and minimize femoral neck shortening. Shu et al. [13] prospectively evaluated the clinical outcomes of 54 patients with FNFs treated by either femoral neck dynamic compression locking system (DCLS), (*n* = 28) or MCCS (*n* = 26). After a mean follow up period of 35.7 months for DCLS group, and 36.7 months for MCCS group, they concluded that DCLS and MCCS were equally effective regarding operation time, incision length, surgical blood loss, and the incidence of perioperative and postoperative complications. However, the DCLS is superior to the MCCS in Harris hip score, fracture healing time, femoral neck shortening, weight-bearing time and fracture healing rate. However, Warschawski et al. [26], retrospectively reviewed 115 patients with non-displaced ICHFs; 81 were treated with standard cancellous cannulated screws (CCS) and 34 with Targon femoral neck (FN) implant (dynamic locking plate); the mean follow-up was 19 and 28 months, respectively. HHS and VAS pain scores weren’t statistical different between both groups. Based on this evidence and the substantial cost difference between the Targon FN and CCS, they suggested CCS for treatment of non-displaced ICHFs.

Analysis of hip range of motion (ROM) revealed significantly improved flexion in the FNLP group, while other movements were comparable between the two groups; this is attributed to enhanced stability of FNLP.

The FNLP group achieved partial and full weight-bearing times significantly earlier than the MCCS group (*p* <.001) indicating superior recovery. Shu et al. [13] reported the partial weight-bearing time and full weight-bearing time in the DCLS group were significantly shorter than those in the MCCS group (*p* =.000).

Radiographic union in this study was significantly faster in the FNLP group (15.2 ± 3.1 weeks) compared to the MCCS group (20.6 ± 2.64 weeks) (*p =*.012). Shu et al. [13] similarly reported the bone healing time of 3.3 ± 0.5months (range, 2.3–4.3months) in the DCLS group was significantly shorter than that of 4.1 ± 0.76months (range, 3.1–6.2months) in the MCCS group (*P* =.000). This reinforces the biomechanical advantage of FNLP in stability and healing.

In this study, complication rates were similar between FNLP and MCCS. Avascular necrosis occurred in 5.4% of FNLP and 2.7% of MCCS cases (*p*˃.999). Nonunion was more frequent in MCCS at three cases (8.1%) than FNLP at one case (2.7%) but was not statistically significant (*p* =.814). Both groups had one case of peri-implant fracture (2.7%), and infection was reported in one MCCS case but none in FNLP (*p*˃.999). Wang et al. [14] reported statistically significant differences in the rates of postoperative nonunion (*p* =.039), shortening of the femoral neck (*p* <.001), and overall complications (*p* =.016) between the two groups of patients in favor of proximal femoral CSLP, they assumed that the major reason for the poorer results in the MCS group was insufficient mechanical stability. Warschawski et al. [26], reported post-operative revision rates and perioperative orthopaedic or non-orthopaedic complications weren’t statistically different between Targon FN (dynamic locking plate) and CCS groups (*p>.999*, =.724 and =.399, respectively).

Several important strengths are present in this study that added to its robustness and reliability. First, the design of the study was a prospective open-labeled RCT, considered a very strong methodology in reducing bias and assuring reliable outcomes. Second, both clinical and radiographic outcomes were evaluated using the most appropriate assessment tools; the functional evaluation using HHS and VAS for pain assessment, and the radiographic evaluation included CT scan for better assessment of fractures. Therefore, the study provides helpful insights to orthopedic surgeons for the determination of the best treatment method.

This study is significantly limited by its short follow-up duration, relatively small sample size and single-centered which may limit the generalizability of findings and reduces the detection of less frequent complications or subgroup differences. It also failed to include important prognostic variables into the analysis of the outcomes such as; bone quality, patient comorbidities, smoking status, and rehabilitation adherence that critically impact the recovery. Larger, multicenter studies with longer follow up that incorporate these variables into the analysis are recommended in the future to validate and expand upon our findings.

## Conclusion

This study concludes that FNLP is superior to conventional MCCS for managing FNF in young adults. FNLP demonstrated significantly better functional outcomes with higher HHS scores, earlier weight-bearing, and faster radiographic union, highlighting its biomechanical stability. MCCS demonstrated significant shorter operative time which is a potential advantage especially in resources-constrained settings. Complication rates were similar between FNLP and MCCS, making MCCS a viable option in selected cases based on fracture severity, surgical expertise, and resources availability. Overall, FNLP is the preferred fixation method for optimal recovery and radiographic outcomes.

## Supplementary Information

Supplementary Material 1.

## Data Availability

The datasets generated and/or analyzed during the current study are available from the corresponding author on reasonable request.

## Abbreviations

FNFs: Femoral neck fractures
FNLP: Femoral neck locking plate
MCCS: Multiple cannulated cancellous screws
MCS: Multiple cancellous screws
HHS: Harris hip score
CT: Computed tomography
RCT: Randomized controlled clinical trial
AVN: Avascular necrosis
FNS: Femoral neck system
VAS: Visual Analogue Scale
CSLP: Cannulated screw locking plate
ICHFs: Intracapsular hip fractures
DCLS: Dynamic compression locking system
